# Subungual Glomus Tumours: Is Magnetic Resonance Imaging or Ultrasound Necessary for Diagnosis?

**DOI:** 10.5704/MOJ.1703.020

**Published:** 2017-03

**Authors:** CR Pandey, N Singh, B Tamang

**Affiliations:** Department of Orthopaedics, Grande International Hospital Kathmandu, Nepal

**Keywords:** glomus tumours, hand, nails, magnetic resonance imaging, ultrasound

## Abstract

**Introduction:**

Diagnosis of subungual glomus tumour is mostly based on detailed history and clinical examination. Recently, Magnetic Resonance Imaging (MRI) and Ultrasound have been proposed as the imaging modality to confirm the clinical diagnosis and in planning the surgical management of these tumours. However, these imaging modalities are not routinely available in rural setting and also are expensive. Due to these limitations, we set out to establish that diagnosis and management of these rare tumours can be based solely on a battery of clinical tests and history taking.

**Materials and Methods:**

Retrospectively, we reviewed nine cases of glomus tumour. A clinical evaluation proforma was developed on the basis of clinical history and specific clinical test for diagnosis of these tumours. All the cases were evaluated and treated surgically by a single surgeon with a specific technique. Post-operatively, diagnosis was confirmed by histopathological examination.

**Results:**

Females (77.78%) were predominantly affected in this series and the tumours commonly occurred in the right hand (66.66%). Spontaneous pain, cold sensitivity test and Love’s Pin test was positive in all cases (100%). Hildreth’s test was positive in 88.89%. In none of the cases the tumours recurred during minimum follow-up of one year. In all cases, histopathological examination confirmed the preoperative diagnosis of glomus tumours.

**Conclusion:**

Diagnosis of glomus tumours can be made clinically based on history taking and clinical examination. Magnetic Resonance Imaging and Ultrasound are not necessary for diagnosis and management of typical subungual tumours.

## Introduction

Glomus tumours are extremely rare benign tumours, accounting for 1% to 4.5% of all hand tumours^1,2^. They are extremely painful tumours believed to be vascular hamartomatous derivatives of the glomus body which is located in the stratum reticulare layer of skin throughout the body. The glomus body is thought to be responsible for thermostasis^3^.

Since the first reported study on glomus tumour by Masson in 1924 ^4^, several studies have surfaced reporting glomus tumours, its pathophysiology and management. One of the most common sites for this tumour is the subungual area, though several other sites have been reported in the literature. In a series of 349 cases of hand tumours, reported by Colon and Upton^5^, nine were found to be glomus tumour.

Glomus tumours are generally diagnosed clinically. However, recent studies emphasize the use of imaging investigations like MRI (Magnetic Resonance Imaging) and ultrasound, to aid in the diagnosis and planning the surgical management^1,6^. Recently, MRI has been proposed as the imaging modality of choice to aid in the diagnosis, along with clinical examination, of these tumours because of its characteristic imaging appearance. MRI is also needed for differentiation of glomus tumours from other soft tissue masses. Despite its usefulness in diagnosing glomus tumours, MRI and other imaging modalities are expensive and require expertise for proper reporting. In addition, MRI evaluation may not be possible due to or lack of availability. Since, clinical history and examination are very characteristic in subungual glomus tumours, we investigated the accuracy and role of clinical history and examination in diagnosis, localization and surgical management of the subungual glomus tumour.

## Materials and Methods

Our study is a retrospective series of nine cases during the period of January 2003 to July 2015. All cases from the medical records (including detailed clinical history, examination, preoperative diagnosis, surgical treatment and histopathological report) maintained and preserved by the operating surgeon, with a preoperative clinical diagnosis of subungual glomus tumours were retrieved. With the exception of antero-posterior and lateral radiographic views of the hand or foot no further imaging modalities were used. In all cases, diagnosis of these tumours was made on the basis of clinical history and examination of the patients presenting with characteristic clinical features and final confirmation was obtained by histopathological examination after surgical excision. Cases of extra-digital glomus tumour were excluded from the study.

All cases included in the study were diagnosed clinically and treated surgically by a single surgeon. A clinical evaluation proforma was developed, based on the patient’s presenting complaints in clinical notes of the surgeon, which was used by co-authors to record the findings. A separate proforma for clinical examination was also developed, based on clinical notes, to aid in the diagnosis of glomus tumours comprising of nail deformity, bluish discoloration of part of nail overlying the tumours, tenderness, Love’s Pin test for localization, Hildreth’s Test and cold sensitivity test^2^. The co-authors recorded the findings from clinical notes. In the proforma, the radiographic details were also recorded.

Love’s Pin test was done by applying pressure to the nail with a pinhead. The area where the pinhead touch induced pain was identified as the region overlying glomus tumours. Hildreth’s test was performed by applying a tourniquet to the affected limb and raising the pressure of the cuff to 250 mm of Hg for upper limb and 350mm of Hg for lower limb. The test was considered positive if there was subsidence in pain with inflation of tourniquet, pin head touch and reappearance of pain with its deflation. Cold sensitivity test was by immersing the hand or foot in cold water. The test was considered positive if there was aggravation of pain on immersing the hand or foot in water. Pre-operative radiographs of hand was taken prior to excision biopsy in order to rule out bony involvement.

**Fig. 1:**
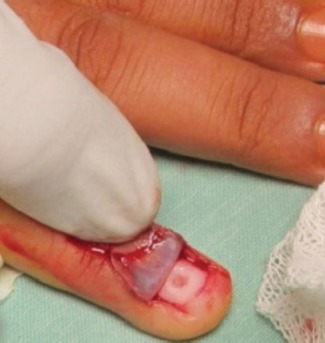
Image of typical glomus tumour in subungual location which was excised by modified Trapdoor technique where nail was lifted proximally and distal attachment was left intact.

**Fig. 2:**
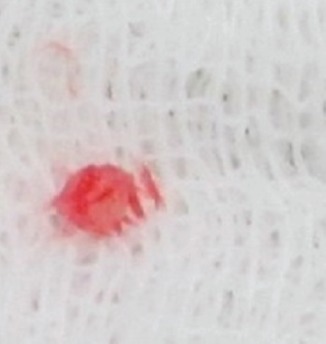
Shows excised Glomus Tumour, excised *in toto* with surrounding normal margin in one of the cases from subungual location.

Digital nerve block anesthesia, under a tourniquet made of a sleeve of glove, was used in all cases. Preoperative localization of the tumour was done by Love’s Pin test before applying tourniquet and administering digital block. The Trap door technique7 with modification (Fig. 1) was used to treat all patients based on the surgeon’s preference of the technique. In this method, the initial steps of localization, anaesthetization and application of a tourniquet remain the same. Parallel incisions to medial and lateral margins of proximal nail fold were given (1 cm). Thereafter, the nail plate was undermined with a nail spatula, starting at the distal free end. Undermining was done from distal to proximal end, until the spatula met the resistance of the onychodermal band. This was followed by carefully lifting the nail plate like a trap door but still maintaining the dorsal pressure on the proximal nail fold to prevent nail plate avulsion. The tumour was excised with size 11 surgical blade. The tumour could be easily identified in all the cases by it being in contrast (darker or deeper red) with rest of the nail bed and matched the localization by Love’s Pin test. The tumour was sharply delineated in all the cases. Careful search was done to identify any satellite lesions in all the cases. The tumours was excised *in toto* with a cuff of surrounding normal tissue of about 1-2 mm (Fig. 2). This was followed by carefully replacing the nail plate back to its normal anatomic position. No suture was used to close the medial and lateral incisions, which were allowed to heal on own. The dressing was changed on the third post-operative day. In all cases, the excised tissue was submitted for histopathological examination and the clinical diagnosis confirmed.

Surgical outcome was evaluated by comparing preoperative and postoperative pain (Love’s Pin test), cold sensitivity test, Hildreth’s test and nail deformity (anatomic alterations, color or trophic changes) at serial follow-ups to rule out the recurrence or incomplete excision of the tumour. Follow-up was done at two weeks, six weeks, six months and at one year post-surgery.

## Results

**Fig. 3:**
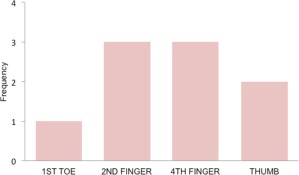
Showing affected digits with their frequency. Bar diagram shows the fingers affected in terms of frequency. Great toe in one case, second and fourth finger in three case each and thumb in two cases were involved.

**Fig. 4:**
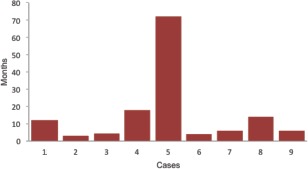
Showing duration of clinical symptoms. Bar diagram shows the duration of clinical symptoms in each case before presentation and diagnosis (in months).

We analyzed, retrospectively, nine cases of glomus tumour which were diagnosed clinically and surgically treated. Confirmation of the diagnosis was done with histopathological examination of the excision biopsy tissue. Seven of the cases were female and two were male. The mean age of the cohort was 36.66 ± 8.90 years (range: 22-53 years). Only tumours occurring in subungual location were included in the study. The right hand was affected in five cases, right great toe in one, and three cases occurred in left hand (Fig. 3). Mean duration of clinical symptoms before presentation and diagnosis was 15.5 ± 21.80 months (range: 3-72 months) (Fig. 4). Summary of the demographic features of the study group is in Table I.

**Table I:** Shows demographic characteristics of the patients with results

Patients	Age (years)	Sex	Side	Pain	Bluish discoloration	Cold sensitivity	Nail changes	Hildreth’s test	Love’s Pin Sensitivity test	Cold Sensitivity test
1	34	M	R	-	-	+	-	+	+	+
2	22	F	R	-	-	+	-	+	+	+
3	36	F	L	-	-	+	-	+	+	+
4	44	F	L	-	-	+	+	-	+	+
5	35	F	R	-	-	+	-	+	+	+
6	53	F	L	-	-	+	-	+	+	+
7	38	F	R	+	+	+	-	+	+	+
8	40	F	R	-	-	+	-	+	+	+
9	28	M	R	-	-	+	-	+	+	+

(M: Male; F: Female; R: Right; L: Left; +: Positive; -: Negative)

Spontaneous pain was present in all the cases along with cold sensitivity (100%). Preoperative bluish discoloration was present in one of the cases. Love’s Pin test localized the lesion in all the cases (100%). Hildreth’s test was positive in eight out of the nine cases (88.89%). Table I shows the clinical symptoms and test results in all the cases individually. No preoperative radiographic changes could be appreciated in any of the cases.

**Fig. 5:**
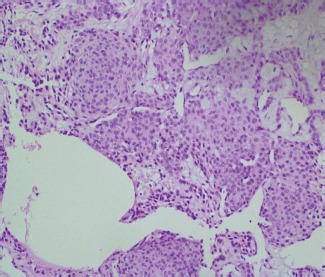
Shows classical Histopathological examination features of Glomus tumour Hematoxylin and eosin staining of glomus shows characteristic compact nests or cords of monotonous rounded or polygonal cells with rounded nuclei.

The postoperative period was uneventful in all cases. Clinical symptoms completely disappeared in all cases and there was no recurrence of symptoms during follow-up. Postoperative recurrence or incomplete excision of the tumour was evaluated by Love’s Pin test, cold sensitivity test and Hildreth’s test. These tests were negative at two weeks, six weeks, six months and one year follow-ups post-surgery signifying no recurrence or incomplete excision. Postoperative nail changes (curving of nail) occurred in one case. Histopathological examination (HPE) in all cases revealed characteristic compact nests or cords of monotonous rounded or polygonal cells with rounded nuclei. The cells had finally granulated chromatin with occasional nuclei without nuclear atypia (Fig. 5).

## Discussion

Wood, in 1812, first described the case of glomus tumour^8^. However, the first histopathological description and the name “glomus” was given by Masson in 1924 ^9,10^. Glomus tumour is present most commonly in subungual location of the fingers. Given the rarity of the tumours and lack of diagnostic criteria, there are chances of misdiagnosis.

A relatively long duration from symptom to diagnosis has been reported in the literature. Yilmaz *et* al^10^, noted in their study an average delay of seven years and four months due to misdiagnosis. They also found that delay was due to treatment with nonsteroidal anti-inflammatory drugs for pain. In our study patients presented with a mean duration of symptom to diagnosis and treatment of 15.5 ± 21.80 months. Recent studies suggest MRI and ultrasound as the radiological tools of choice to aid in diagnosis of glomus tumour^1,6^. However, studies by Llanos *et al*^11^ and Matloub *et al*^12^, reported that MRI and ultrasound do not provide specific findings, though they are accurate in locating and predicting the size of the tumours. Since these investigations are expensive and not readily available in lower socioeconomic countries, our study tried to evaluate a specific diagnostic tool for glomus tumour based on clinical examination and history of the symptoms.

Glomus tumour, as mentioned in several studies, has been characteristically described by severe pain, point tenderness, and cold sensitivity. Yilmaz *et al*^10^ noted that all patients had localized tenderness and spontaneous pain in their case series. Van Geertruyden *et al*^4^, in their series of 51 patients noted spontaneous pain in 80%, touch sensitivity in 100% and cold sensitivity in 63%. Giele^13^ noted the sensitivity and specificity of Hildreth’s test to be 92% and 91% respectively in detection of glomus tumour in their series of 24 patients with hand tumours. Netscher *et al*^14^, in their study, found cold sensitivity test to be 100% sensitive, specific and accurate whereas Love’s pin test to be 100% sensitive and 78% accurate. Also, Hildreth’s test was 71.4% sensitive, 100% specific and 78% accurate. These studies support that clinical history and physical examination is a reasonable way of diagnosing subungual glomus tumours. Based on these studies, we combined the clinical features as shown in Table I and found that all cases of subungual glomus tumour could be diagnosed on the basis of the above mentioned clinical features. However, we did not include the trans-illumination test which is 23 to 38% sensitive and 90% specific^14^.

We also tried to evaluate the surgical treatment success if only clinical history and examination were taken into consideration for diagnosis and planning treatment. Localization was performed with Love’s Pin test and surgery was planned accordingly to completely excise the lesion with a normal cuff of surrounding tissue, approximately 1-2 mm in all dimensions. We did not have any recurrences in any of the cases at subsequent follow-ups. Thus, our study supports the findings of Drape *et al*^9^ that complete excision of the tumour with capsule decreases the chance of recurrence. Therefore, MRI is not needed for solitary lesions in planning treatment as well and with planned surgical excision with a normal cuff of tissue, recurrence can be minimized.

Limitation of our study was the relatively small number of cases but keeping in mind the rarity of the tumours, the number mentioned above can be used to derive conclusions. Also, as with any other retrospective case series, the chances of missing a case if it was not maintained in records, could not be ruled out. Thus, studies with larger patient numbers and preferably prospective in nature is needed in future.

## Conclusion

We conclude that MRI or ultrasound is not necessary in case of classical subungual glomus tumour and clinical history and examination are sufficient in diagnosing and planning the management. However, MRI and ultrasonography have a definite role when there are multiple lesions and when there is uncertainty regarding the diagnosis with lesions in unusual locations.

## Conflict of Interest

The authors declare that none of them received any financial assistance from any institution, person or company for this study. There is no potential conflict of interest.
